# Unnecessity of Routine Dissection of Right Central Lymph Nodes in cN0 Papillary Thyroid Carcinoma Located at the Left Thyroid Lobe

**DOI:** 10.3389/fonc.2021.685708

**Published:** 2021-07-01

**Authors:** Songtao Zhang, Runfang Zhang, Chao Wang, Wenbo Gong, Chen Zheng, Qigen Fang, Liyuan Dai

**Affiliations:** Department of Thyroid and Head Neck, Affiliated Cancer Hospital of Zhengzhou University, Henan Cancer Hospital, Zhengzhou, China

**Keywords:** LN-prRLN, cN0, papillary thyroid carcinoma, left thyroid lobe, lymph node metastasis

## Abstract

**Objective:**

The lymph node posterior to the right recurrent laryngeal nerve (LN-prRLN) is an important part of the central lymph nodes (LNs). We aimed to explore the rate and predictors of LN-prRLN metastasis in cN0 papillary thyroid carcinoma (PTC) located at the left thyroid lobe.

**Methods:**

Patients with surgically treated primary left lobe PTC were retrospectively enrolled. The metastatic distribution of LN-prRLN and postoperative complications were assessed. The association between LN-prRLN metastasis and clinicopathological variables was evaluated by univariate and multivariate analyses.

**Results:**

A total of 857 patients were included for the analysis. Central LN metastasis was noted in 310 (35.3%) cases. The most (27.6%) and least (1.7%) commonly involved LNs were the left paratracheal LN and the LN-prRLN. In the univariate analysis, the tumor size, multifocality, the extent of extrathyroidal extension (none *vs.* macroscopic *vs.* macroscopic maximal), and perineural invasion were associated with positive LN-prRLN. In the multivariate analysis, tumor size of >40.0 mm and macroscopic maximal invasion were found as the only two independent predictors. Transient and permanent hypoparathyroidism were noted in 90 (10.2%) and 13 (1.5%) patients, respectively. Voice change was noted in 40 (4.6%) patients, and 20 patients recovered.

**Conclusions:**

In cN0 PTC located at the left lobe, LN-prRLN metastasis was very uncommon. We found that LN-prRLN dissection is not required routinely, but should be performed if the tumor size is >40.0 mm and macroscopic maximal extrathyroidal extension is present.

## Introduction

Papillary thyroid carcinoma (PTC) is the most common thyroid malignancy, accounting for >85% of all thyroid cancers (1). Central neck lymph node (LN) metastasis is frequent even in cN0 PTC, leading to a considerable number of patients with unilateral cN0 disease developing contralateral central lymph node metastasis. Usually, central neck LNs consist of four groups: prelaryngeal LNs, pretracheal LNs, left paratracheal LNs, and right paratracheal LNs. Owing to the anatomical difference of recurrent laryngeal nerves of both sides, the right paratracheal LNs are divided into lymph nodes posterior to the right recurrent laryngeal nerve (LN-prRLN) and those anterior to the right recurrent laryngeal nerve (LN-arRLN).

Currently, both the American Thyroid Association and the Chinese Thyroid Association guidelines have stressed the importance of central neck dissection (CND) in PTCs (2, 3). However, both the guidelines do not clarify the extent of LN-prRLN dissection in central lymphadenectomy. Also, there is a controversy regarding the resection of LN-prRLN in cN0 PTC. A number of researchers have tried to analyze possible predictors of LN-prRLN metastasis. The commonly accepted risk factors include right thyroid lobe tumor, tumor size of ≥1 cm, multifocality, capsular invasion, extrathyroidal invasion (ETE), lateral neck LN metastasis, and LN-arRLN metastasis (4). However, some patients have LN-prRLN metastasis with PTC located at the left thyroid lobe (5–7). Unfortunately, further details are not available in this regard; therefore, in this study, we aimed to analyze the incidence and possible predictors of LN-prRLN metastasis in PTC located at the left thyroid lobe.

## Patients and Methods

### Ethics

Our hospital institutional research committee approved this study, and all participants signed an informed consent agreement. All procedures performed were in accordance with the ethical standards of the institutional and/or national research committee and the 1964 Helsinki Declaration and its later amendments or comparable ethical standards.

### Patient Selection

From January 2018 to January 2021, medical records of adult (>18 years) patients with surgically treated primary PTC were retrospectively reviewed. The enrolled patients fulfilled the following inclusion criteria: the diagnosis of PTC was confirmed by postoperative pathology, the neck status was defined as cN0, there were no suspicious malignant nodules located at the thyroid right lobe or the isthmus by preoperative ultrasound, and bilateral CND was performed. The exclusion criteria were: a history of neck surgery, a family history of cancer, and a history of neck radiotherapy. Clinical data of the enrolled patients [demography; pathology, including ETE; capsular invasion; perineural invasion (PNI); and lymphovascular invasion (LVI), BRAF 600E mutation; and postoperative complications] were extracted and analyzed.

### Definitions of Important Variables

A left lobe PTC was defined as a tumor having a medial boundary without involving the lateral border of the trachea. A cN0 neck was defined according to the following ultrasound features: the absence of an echogenic hilum, round shape, microcalcification, peripheral blood flow on color Doppler images, and cystic changes (8); and the following computerized tomography (CT) features: area with clear evidence of non-fat, low-density, or liquid components; largest diameter of >15 mm at level II and >10 mm at other levels; and a ratio of the longest to the smallest diameter of ≤2 (9). The tumor size was defined as the longest diameter of the tumor. Capsular invasion was defined as a tumor invading the thyroid capsular. ETE was defined as a tumor invading the structures outside the thyroid and was divided into two groups: macroscopic ETE (invading into the anterior cervical muscle) and macroscopic maximal ETE (invading into the trachea, larynx, and the recurrent laryngeal nerve). Both capsular invasion and ETE were evaluated according to the intraoperative finding and postoperative pathology.

### Surgical Treatment Proposal

According to the guideline of the Chinese Thyroid Association, three surgical procedures (unilateral or sub-total or total thyroidectomy with CND) are available. The selection of the method was dependent on the systemic consideration of the patient’s condition and the surgeon’s preference. The surgical specimens of the central LNs were usually divided into five subgroups, including prelaryngeal LN, pretracheal LN, left paratracheal LN, LN-arRLN, and LN-prRLN, and were sent for pathological examination individually.

### Statistical Analyses

The association between clinicopathological variables and LN-prRLN metastasis was assessed using the chi-square test. A univariate analysis was performed; factors that were significant in the univariate analysis were analyzed in the multivariate analysis to find the independent predictors. All statistical analyses were performed using SPSS version 20.0, and a p-value of <0.05 was considered statistically significant.

## Results

### Baseline Data

A total of 879 patients were enrolled for analysis. The mean age of the patients was 48.5 (range, 19−74) years. There were 634 (72.1%) female and 245 (27.9%) male patients. The mean tumor size was 10.0 (range, 2.1−55.8) mm. Coexistent Hashimoto’s thyroiditis developed in 325 (37.0%) patients. A total of 217 (24.7%) patients had two malignant nodules, and 88 (10.0%) had three or more malignant nodules. Capsular invasion was present in 175 (19.9%) patients. PNI and LVI developed in 80 (9.1%) and 76 (8.6%) patients, respectively, and 689 (78.4%) patients had BRAF 600E mutation. Macroscopic and macroscopic maximal ETE were found in 100 (11.4%) and 26 (3.0%) patients, respectively. Total, sub-total, and unilateral thyroidectomy were performed in 157 (17.9%), 308 (35.0%), and 414 (47.1%) patients, respectively (**Table 1**).

**Table 1:** Clinical and pathologic data of the enrolled 879 patients.

Variables	Number (%)
Age	
<55	489 (55.6%)
≥55	390 (44.4%)
Gender	
Male	245 (27.9%)
Female	634 (72.1%)
Tumor size	
0–5.0 mm	187 (21.3%)
5.0–10.0 mm	395 (44.9%)
10.0–40.0 mm	205 (23.3%)
>40.0 mm	92 (10.5%)
Coexistent Hashimoto’s thyroiditis	325 (37.0%)
Multifocality	
1	574 (65.3%)
2	217 (24.7%)
>2	88 (10.0%)
Capsular invasion	175 (19.9%)
Extrathyroidal invasion	
Macroscopic	100 (11.4%)
Macroscopic maximal	26 (3.0%)
Perineural invasion	80 (9.1%)
Lymphovascular invasion	76 (8.6%)
BRAF 600E mutation	689 (78.4%)
Operation type	
Total thyroidectomy+central neck dissection	157 (17.9%)
Sub-total thyroidectomy+central neck dissection	308 (35.0%)
Unilateral lobectomy+central neck dissection	414 (47.1%)
Hypoparathyroidism	
Transient	90 (10.2%)
Permanent	13 (1.5%)
Vocal cord paralysis	
Transient	40 (4.6%)
Permanent	35 (4.0%)

### Central Neck LN Metastasis

Positive central LN was seen in 310 (35.3%) patients. The most common metastatic pattern was isolated metastasis of left paratracheal LN, and the least common metastatic pattern was the simultaneous metastasis of left paratracheal LN, LN-arRLN, and LN-prRLN. The most frequently involved LN was the left paratracheal LN (243 cases, 27.6%), followed by prelaryngeal LNs (96 cases, 10.9%) and pretracheal LNs (94 cases, 10.7%). The least common involved site was the LN-prRLN (15 cases, 1.7%). There was no skip metastasis of LN-prRLN (**Table 2**).

**Table 2:** Lymph node (LN) metastasis pattern in the 310 patients with positive central lymph nodes.

Metastatic sites	Number (%)
Left paratracheal LN	134 (43.2%)
Prelaryngeal LN	67 (21.6%)
Left paratracheal LN+Pretracheal LN	45 (14.5%)
Left paratracheal LN+Pretracheal LN+Prelaryngeal LN	29 (9.4%)
Left paratracheal LN+Pretracheal LN+LN-arRLN*	20 (6.5%)
Left paratracheal LN+LN-arRLN+LN-prRLN^#^	15 (4.8%)

*LN-arRLN, lymph node anterior to the right recurrent laryngeal nerve.

^#^LN-prRLN, lymph node posterior to the right recurrent laryngeal nerve.

### Association Between LN-prRLN Metastasis and Clinicopathological Variables

In the univariate analysis, 10 (10.9%) of 92 patients with a tumor size >40.0 mm had LN-prRLN metastasis, and it was significantly higher than that in patients with a tumor size <40.0 mm (p<0.001). The positive LN-prRLN rates in patients with one, two, and more than two malignant nodules were 0.7%, 2.3%, and 6.8%, respectively (p=0.001). A total of 4% and 15.4% of patients with macroscopic and macroscopic maximal ETE had LN-prRLN metastasis, respectively, compared to 7 (0.9%) of 753 patients without ETE (p<0.001). Eighty patients had PNI, 5.0% of whom showed LN-prRLN metastasis; it was significantly higher than the 1.4% of patients without PNI (p=0.040). No apparent associations between LN-prRLN metastasis and other variables were noted (all p>0.05) (**Table 3**).

**Table 3:** Univariate analysis of the predictors of lymph node posterior to the right recurrent laryngeal nerve (LN-prRLN) metastasis.

Variables	LN-prRLN status		p
	Positive (n = 15)	Negative (n = 864)	
Age			
<55	6 (1.2%)	483 (98.8%)	
≥55	9 (2.3%)	381 (97.7%)	0.219
Gender			
Male	5 (2.1%)	240 (97.9%)	
Female	10 (1.6%)	624 (98.4%)	0.772
Tumor size			
0–5.0 mm	0	187 (100%)	
5.0–10.0 mm	1 (0.3%)	394 (99.7%)	
10.0–40.0 mm	4 (2.0%)	201 (98.0%)	
>40.0 mm	10 (10.9%)	82 (89.1%)	<0.001
Coexistent Hashimoto’s thyroiditis			
Yes	7 (2.2%)	318 (97.8%)	
No	8 (1.4%)	546 (98.6%)	0.433
Multifocality			
1	4 (0.7%)	570 (99.3%)	
2	5 (2.3%)	212 (97.7%)	
>2	6 (6.8%)	82 (93.2%)	0.001
Capsular invasion			
No	9 (1.3%)	695 (98.7%)	
Yes	6 (3.4%)	169 (96.6%)	0.058
Extrathyroidal invasion			
No	7 (0.9%)	746 (99.1%)	
Macroscopic	4 (4.0%)	96 (96.0%)	
Macroscopic maximal	4 (15.4%)	22 (84.6%)	<0.001
Perineural invasion			
No	11 (1.4%)	788 (98.6%)	
Yes	4 (5.0%)	76 (95.0%)	0.040
Lymphovascular invasion			
No	12 (1.5%)	791 (98.5%)	
Yes	3 (3.9%)	73 (96.1%)	0.133
BRAF 600E mutation			
No	4 (2.2%)	175 (97.8%)	
Yes	11 (1.6%)	689 (98.4%)	0.748
Operation type*			
TT+central neck dissection	4	153	
ST+central neck dissection	5	303	
UL+central neck dissection	6	408	0.658

*TT, total thyroidectomy; ST, sub-total thyroidectomy; UL, unilateral lobectomy.

In the multivariate analysis, there were only two independent predictors of tumor size and ETE. There was no additional risk of LN-prRLN metastasis until the tumor size increased to 40.0 mm. The possibility of LN-prRLN metastasis increased by 1.5-fold when the tumor size was >40.0 mm. The presence of macroscopic maximal ETE was associated with a nearly 3.7-fold increased risk of LN-prRLN metastasis (**Table 4**).

**Table 4:** Multivariate analysis of the predictors of lymph node posterior to the right recurrent laryngeal nerve metastasis.

Variables	p	OR [95% CI]
Tumor size		
0–5.0 mm		
5.0–10.0 mm	0.547	1.335 [0.356–2.001]
10.0–40.0 mm	0.098	1.889 [0.943–3.542]
>40.0 mm	0.012	2.337 [1.245–5.987]
Multifocality		
1		
2	0.452	1.766 [0.463–3.226]
>2	0.067	2.117 [0.970–6.338]
Extrathyroidal invasion		
No		
Macroscopic	0.156	2.116[0.768–3.574]
Macroscopic maximal	<0.001	4.763 [2.456–8.448]
Perineural invasion	0.261	2.000 [0.756–4.774]

### Postoperative Complications

Transient and permanent hypoparathyroidism were noted in 90 (10.2%) and 13 (1.5%) patients, respectively. Voice change developed in 40 (4.6%) patients, of whom 20 had consistent dysfunction with vocal cord paralysis. Twenty patients recovered, but only 5 regained normal vocal cord activity (**Table 1**).

### Oncologic Outcome

During our follow-up, 43 (4.9%) patients received adjuvant iodine 131 therapy. Lateral and central neck lymph node metastases were noted in 15 (1.7%) and 18 (2.0%) patients, respectively. They all underwent a second operation. No patient died.

## Discussion

The most important finding of the current study was that the LN-prRLN metastasis rate was very low; it only affected 1.7% of patients with PTC located at the left thyroid lobe. The independent predictors of LN-prRLN metastasis were a tumor size of >40.0 mm and macroscopic maximal ETE. Routine LN-prRLN dissection was not required in patients with PTC located at the left thyroid lobe; however, it should be performed if the tumor size is >40.0 mm and macroscopic maximal ETE is present.

The importance of LN-prRLN was first mentioned by Grodski et al. (10); the authors opined that although there is an increased possibility of nerve injury owing to traction and elevation, the removal of LN-prRLN is usually required in CND. Lee et al. (6) were probably the first to explore the significance of LN-prRLN. They reported that among 11.4% of 123 PTC patients who had positive LN-prRLN, metastasis was more likely in PTCs at the right lobe, if the tumor size was >1.0 cm or a great number of positive central LNs were detected, or if lateral LN metastasis was detected. Unfortunately, further multivariate analysis was not performed; hence, the influence of confounding factors could not be evaluated. Bae et al. (11) retrospectively enrolled 369 PTC patients undergoing therapeutic or prophylactic CND and found positive LN-prRLN in 12.2% of the patients. Both univariate and multivariate analyses showed that a tumor size of >1 cm and the presence of three or more positive LNs were significantly correlated with LN-prRLN metastasis. A slightly higher rate (16.6%) was reported by Kim et al. (12); the possible explanation is that only right-sided PTC was included in this study. Further, the authors have described that females did not tend to have LN-prRLN metastasis, but central LN metastasis and lateral LN metastasis were independently associated with an increased risk of LN-prRLN metastasis. Yuan et al. (13) had reported the highest incidence of 38.3% of LN-prRLN metastasis, but this study only included 81 PTC patients. In the univariate analysis, there were five significant variables of lateral LN metastasis, viz. LN-arRLN metastasis, pathological tumor size, capsular invasion, and ETE. However, in the multivariate analysis, LN-arRLN metastasis was the only independent predictor for positive LN-prRLN. Similar findings were reported by Zhang et al. (14), Li et al. (15), and Liu et al. (16). However, cN+ patients were analyzed together with cN0 patients in these studies, although in routine clinical practice, LN-prRLN dissection is always performed for a cN+ neck. Hence, more attention should be paid to cN0 PTC.

To the best of our knowledge, only two studies had focused on cN0 patients. Ito et al. (17) included 922 patients with PTC located at the right lobe and showed that 14% of patients had LN-prRLN metastasis. Further, a tumor size of ≥2 cm and ETE were independently associated with positive LN-prRLN. Li et al. (18) reported that 8.3% of 338 patients had LN-prRLN metastasis. Several factors, such as age, gender, tumor size, and capsular invasion, were associated with the presence of LN-prRLN metastasis. In the multivariate analysis, female patients with no capsular invasion were least likely to have positive LN-prRLN. The difference in the incidence of LN-prRLN metastasis between the two studies was mainly attributed to the difference in the study subjects.

Li et al. (18) stated that LN-prRLN metastasis could develop in patients with PTC located at the left lobe. Similar findings were reported by the other authors (5, 13, 14). However, the exact incidence and potential predictors of LN-prRLN metastasis were never analyzed. The incidence of LN-prRLN metastasis in PTC located at the left lobe was extremely low, and it developed only when there were positive paratracheal LNs. This suggests that there is no necessity for routine LN-prRLN dissection in cN0 PTC located at the left lobe. Further, the metastasis rate in our study was apparently lower than those reported in previous studies (5–18); the possible explanation is the relatively long distance between the primary site and LN-prRLN in our study.

The risk factors for LN metastasis were frequently analyzed, and tumor size was identified as one of the most important predictors. Ito et al. (19) reported that 73% of 1593 cN0 PTC patients with a tumor size >2 cm PTC had LN metastasis, and positive LNs only developed in 55% of 1626 patients with a tumor size ≤2 cm. The difference was significant, and the odds ratio for the tumor size in the multivariate analysis was greater than those for age, gender, multiplicity, and ETE. Other studies also confirmed the association between the tendency of LN metastasis and tumor size (>1 cm *vs.* >2 cm) (5–18). Larger tumors were more likely to behave aggressively. Contralateral central LN metastasis was not uncommon in some unilateral cN0 PTCs, the incidence rate being 3.88−30.63% (20, 21). In a meta-analysis that included 1,370 patients from seven studies, the incidence of contralateral central LN metastasis was 18.21% in patients with a tumor size ≥1 cm, and it was significantly higher than 7.59% in patients with a tumor size <1 cm. Unfortunately, this study did not specify the subgroups of right parathyroid LNs and also did not present the outcomes if the tumor size was ≥40 mm. We first noted that the incidence of LN-prRLN metastasis remained unchanged until the tumor size was >40.0 mm. This was supported by the metastatic pattern. This finding reflected the fact that isolated metastasis of LN-prRLN is rare and that, in most instances, positive LN-arRLN emerges first followed by the possible emergence of LN-prRLN metastasis (5–10). Additionally, the overall positive right paratracheal LN rate in our study was as low as 4.0%, but it increased to 21.7% if the tumor size was greater than 40.0 mm. Then our finding would suggested dissection of LN-prRLN and LN-arRLN was not required until the tumor size in left lobe was >40.0 mm.

ETE was another important variable affecting LN metastasis and disease recurrence in PTC. Its extent was categorized as macroscopic invasion, if invasion was limited to only strap muscles, and macroscopic maximal invasion, if invasion extended beyond the strap muscles (22). In accordance with the American Joint Committee on Cancer staging system, irrespective of the tumor size, patients with ETE were classified into T3 or T4. Thus, ETE represents one of the main characteristics of an advanced primary tumor, and these patients are supposed to undergo more radically surgical treatment; furthermore, the presence of ETE is usually associated with reduced disease-free survival (2, 23, 24). Sun et al. (20) reviewed 1,746 patients from 9 studies and found PTC showing ETE had a contralateral central LN metastasis rate of 13.1%, compared to 7.9% in PTC without ETE, having a significant difference. The finding was confirmed by a subsequent meta-analysis (23). However, these studies regarded macroscopic and macroscopic maximal ETE as a single variable. Liu et al. (25) compared the differences in clinical outcomes according to the presence and extent of ETE between different primary tumor size groups. They found that among PTC patients with primary tumors >1 cm, those with ETE had a higher recurrence rate than those without ETE. Also, only macroscopic maximal ETE affected recurrence in patients with PTC >1 cm size. Compared to macroscopic ETE, the macroscopic maximal ETE had a higher risk of LN metastasis. Similarly, Kim et al. (26) noted that the odds ratio of macroscopic maximal ETE was nearly three times higher than that of macroscopic ETE for pathological lymph node metastasis; furthermore, the ETE extent was related to differences in recurrence-free survivals. The authors indicated that macroscopic ETE was independent of macroscopic maximal ETE; however, all other previous studies evaluating the predictors of LN-prRLN metastasis did not take this concept into consideration (5–8). This study is, thus, the first to analyze the individual effects of these two factors. We found that, unlike macroscopic ETE, macroscopic maximal ETE was significantly associated with LN-prRLN metastasis. Macroscopic maximal ETE was easily detected during surgery, and its presence could be used to guide surgical extent in the neck. Our results imply that if there is a macroscopic maximal ETE, the LN-prRLN should be completely extirpated with combined neck dissection in the central neck to remove any nodal diseases having potentially adverse pathological features.

Vocal cord paralysis and hypoparathyroidism were the two of the most serious complications after thyroid surgery. These could significantly compromise the patient’s quality of life. Bae et al. (11) noted that 7.0% and 10.8% of 369 patients undergoing LN-prRLN dissection had postoperative voice change and required calcium supplementation for 6 months postoperatively, respectively. Yuan et al. (13) reported that among 81 PTC patients with LN-prRLN excision, transient recurrent laryngeal nerve palsy and hypoparathyroidism were found in 7.4% and 51.9% of patients, respectively. There was no permanent dysfunction. Similar findings were reported by the other authors (16–18, 27) and us, thus reinforcing the need for LN-prRLN dissection to be performed by an experienced surgeon.

Limitation in current study must be acknowledged, this study was retrospective, then our results were significantly influenced by the surgical procedures, therefore, more high quality prospective researches were need to clarify the important issue; there was over treatment in some patients, but we hope this research could improve our treatment principle to better serve the patients.

In summary, in cN0 PTC located at the left lobe, LN-prRLN metastasis was very uncommon. Routine LN-prRLN dissection is not required but should be performed if the tumor size is >40.0 mm and ETE is present.

## Data Availability Statement

The original contributions presented in the study are included in the article/supplementary material. Further inquiries can be directed to the corresponding author.

## Ethics Statement

The studies involving human participants were reviewed and approved by Our hospital (Henan Cancer Hospital) institutional research committee approved this study. The patients/participants provided their written informed consent to participate in this study.

## Author Contributions

All authors contributed to the article and approved the submitted version.

## Conflict of Interest

The authors declare that the research was conducted in the absence of any commercial or financial relationships that could be construed as a potential conflict of interest.
